# Effect of Non-tuberculous Mycobacteria on Host Biomarkers Potentially Relevant for Tuberculosis Management

**DOI:** 10.1371/journal.pntd.0003243

**Published:** 2014-10-16

**Authors:** S. Dhanasekaran, Synne Jenum, Ruth Stavrum, Harald G. Wiker, John Kenneth, Mario Vaz, T. Mark Doherty, Harleen M. S. Grewal, TB Trials Study Group

**Affiliations:** 1 Department of Clinical Science, Faculty of Medicine and Dentistry, University of Bergen, Bergen, Norway; 2 Center for Immune Regulation, Rikshospitalet- Radium Hospitalet Medical Centre, University of Oslo, Oslo, Norway; 3 Division of Infectious Diseases, St. John's Research Institute, Koramangala, Bangalore, India; 4 Division of Health & Humanities, St. John's Research Institute, Koramangala, Bangalore, India; 5 GlaxoSmithKline Pharma, Vaccines, Brøndby, Denmark; 6 Department of Microbiology, Haukeland university hospital, University of Bergen, Bergen, Norway; University of Tennessee, United States of America

## Abstract

**Background:**

Non-tuberculous mycobacteria (NTM) are different from *Mycobacterium tuberculosis* (MTB) both in their ubiquitous environmental distribution and in their reduced capacity to cause disease. While often neglected in favour of other infectious diseases, NTM may interfere with important aspects of TB control and management, namely the efficacy of new anti-tuberculosis (TB) vaccines; the immuno-diagnostic Tuberculin skin test (TST) and QuantiFERON TB Gold In Tube assay (QFTGIT); and immune biomarkers explored for their diagnostic and/or predictive potential. Our objective was therefore to explore host immune biomarkers in children who had NTM isolated from respiratory and/or gastric specimens.

**Methodology and Principle Findings:**

The present study was nested within a prospective cohort study of BCG-vaccinated neonates in Southern India. In this setting, immune biomarkers from peripheral blood were analyzed in 210 children aged <3 years evaluated for TB using dual-colour-Reverse-Transcriptase-Multiple-Ligation-dependent-Probe-Amplification (dcRT-MLPA) and Bio-Plex assays. The children were classified based on clinical examination, chest X-rays and mycobacterial culture reports as either: 1) TB disease, 2) NTM present and 3) controls. The study shows a down-regulation of *RAB33A* (p<0.001) and up-regulation of *TGFβ1*, IL-2 and IL-6 (all p<0.05) in children with TB disease, and that *RAB33A, TGFBR2* and IL-10 (all p<0.05) were differentially expressed in children with NTM present when compared to children that were culture negative for MTB and NTM (controls).

**Conclusions and Significance:**

Carriage of NTM may reduce the specificity of future diagnostic and predictive immune biomarkers relevant to TB management.

## Author Summary

Non-tuberculous mycobacteria (NTM) are a ubiquitous group of mycobacteria found in the environment. They are opportunistic pathogens causing human disease, especially in immunocompromised individuals. Differentiation between NTM infection and tuberculosis (TB) can be difficult. Data on incidence of NTM in TB endemic countries is limited due to resource intensive methods required for identification and a considerable workload due to other diseases. The present study was based on children investigated for TB and classified according to chest X-rays and mycobacterial culture reports. We explored host immune biomarkers which are potentially relevant to TB management, in children with confirmed NTM exposure. The findings from the present study suggest that NTM exposure modulates TB-relevant immune biomarkers in the host by eliciting some of the same immune responses as MTB infection. This is may be of importance when evaluating immunological correlates of protection in the setting of TB vaccine trials and potential TB diagnostic biomarkers.

## Introduction

Non-tuberculous mycobacteria (NTM) are widely distributed in soil and water [1]. The innumerable species comprising the genus *Mycobacterium* have differences in pathogenicity, virulence, response to drugs, *in-vivo* adaptation and growth characteristics [2]. Pathogens of the genus *Mycobacteria* are responsible for serious human diseases, including tuberculosis (TB) and leprosy. However, the host-pathogen interactions during atypical (non-tuberculous) mycobacterial infection remain poorly characterized [3], [4]. In recent years, NTM infection is recognized to play a role in exacerbation of chronic pulmonary disorders, e.g. cystic fibrosis and chronic obstructive pulmonary disease and the cause of TB-like disease in the immunocompromised [5]. The data on the prevalence of NTM in TB-endemic countries is limited. The probable factors for under-reporting of NTM are lack of: awareness, standardized or accepted criteria to define NTM respiratory disease and laboratory infrastructure to identify NTM [2].

Furthermore, in the context of TB, the background prevalence of NTM is discussed [6], as one of the factors explaining the variable efficacy of the BCG vaccine in clinical trials (0–90%) [7]. Subjects with high purified protein derivative (PPD)-specific IFN-γ responses (from NTM exposure) prior to BCG-vaccination have reduced PPD-specific IFN-γ responses post-vaccination compared to subjects with lower responses pre-vaccination, suggesting an inhibition of BCG efficacy by prior NTM exposure [6], [8]. Inhibition of BCG efficacy by prior exposure to NTM has also been demonstrated *in vivo* in animal models [9]. These findings indicate that NTM exposure affects anti-mycobacterial host immune responses raising the possibility of interference with novel immune read-outs, explored for their potential as new diagnostics or immune-correlates of protection from TB progression [10], [11]. Unraveling these aspects are important, given that new diagnostics are needed, particularly in populations with a high proportion of unconfirmed TB cases, such as young children [12] and in immunocompromised subjects the latter having an increased risk of NTM-related disease. Immune-correlates of protection from TB progression are also needed for vaccine efficacy trials and targeted preventive treatment of subjects latently infected with *M. tuberculosi*s (MTB). Furthermore, the presence of NTM likely interferes with the established immuno-diagnostic methods: the Tuberculin skin test (TST) and the QuantiFERON TB Gold in tube assay (QFTGIT), both of which demonstrate a varying degree of cross-reaction with a limited number of NTM species [13]. A recent study from a TB-endemic country shows that NTM were isolated in 6% of all children investigated for pulmonary TB [14]. In our previous study evaluating diagnostic immune biomarkers for MTB infection and disease in young children, the high prevalence of NTM isolates in clinical specimens (from ∼30% children without TB disease) made us query to what extent the presence of NTM may mask biomarker differences between children with TB disease, MTB infection and MTB uninfected controls [15]. To our knowledge, immune responses in children in a TB endemic setting with NTM exposure have not been previously characterized.

Based on these knowledge gaps, our objective was therefore to explore immune responses in children with NTM isolated from respiratory and/or gastric specimens. In the setting of a longitudinal cohort study of BCG-vaccinated neonates in southern India, we analyzed a pre-selected panel of transcriptional and translational biomarkers in 210 children evaluated for TB and classified according to their chest X-ray (CXR) and mycobacterial culture reports. The immune biomarkers in children with NTM present were compared with responses in children that were culture negative for MTB and NTM (controls) and children with TB disease but without NTM present (TB patients). Initially, children with NTM present were analyzed regardless of their TST and QFTGIT results and subsequently reanalyzed based on responses to these tests, in order to determine to what extent the results were modulated by latent MTB infection.

## Materials and Methods

### Study details and classification of study subjects

The sample collection and study design have been described in detail elsewhere [15]. Briefly, 4382 neonates all BCG-vaccinated within 72 hours of delivery were enrolled within 2 weeks of birth following parental consent. The study was conducted at the Palamaner Taluk, Chittoor district, Southern India. The recruited children were randomly (based on the population units where they were born) assigned to active (visited bimonthly; to check for recent TB contact, symptoms and anthropometry; N = 2215) and passive (TB education given to parents/guardian but with no scheduled home visits; N = 2167) surveillance arms, and monitored at fixed time points as outlined in the study protocol for 2 consecutive years. During the study period, 746 children were referred to a TB case verification ward (CVW) on suspicion of TB. Referral criteria were 1) respiratory symptoms suggestive of TB (cough ≥2 weeks), failure to thrive (FTT) defined as any of the following; (a) unexplained weight loss or no weight-gain for two consecutive visits; (b) downward crossing of two percentile lines on the weight-for-age growth chart or (c) weight persistently tracking below the 3^rd^ percentile of weight for age growth chart 2) a history of known TB exposure or 3) a TST ≥10 mm at study closure. The diagnostic assessment included: clinical examination, a CXR anteroposterior view, two induced sputa (IS) and gastric aspirates (GA) on consecutive days (for smear and culture), TST (2 TU/0.1 mL of PPD RT-23; Span Diagnostics, Ltd., Bangalore, India) and QFTGIT (Cellestis Inc, Valencia, California, USA). IS and GA samples were examined by fluorescent microscopy (Auramine) and culture using liquid (Mycobacterial Growth Indicator Tube) and solid (Löwenstein-Jensen) medium [16]. Positive cultures were confirmed by the HAIN kit (GenoType MTBC, Hain Life Sciences, Germany). Direct PCR (The COBAS TaqMan MTB Test, Roche) was undertaken on culture negative specimens for infants with CXR findings suggestive of TB.

From the 746 children investigated at the CVW, the 210 children included in this study were originally selected for an exploratory study of biomarkers with a diagnostic potential in young children assessed for TB disease and MTB infection. All children with clinical TB disease (n = 13) were included. They were diagnosed by the identification of MTB in culture or by PCR (Roche PCR test) (n = 4) or; in the case of cultures negative for MTB and NTM, by pathology consistent with TB at CXR as judged by 2/3 radiologists (n = 9). Children without TB disease (normal CXR and culture negative for MTB), but presumed to be infected based on positive results for TST and/or QFTGIT (n = 90), were also included. In addition, gender matched MTB uninfected controls (normal CXR and culture negative for MTB, TST and QFTGIT negative; n = 107) were selected amongst other investigated children.

**Figure 1:**
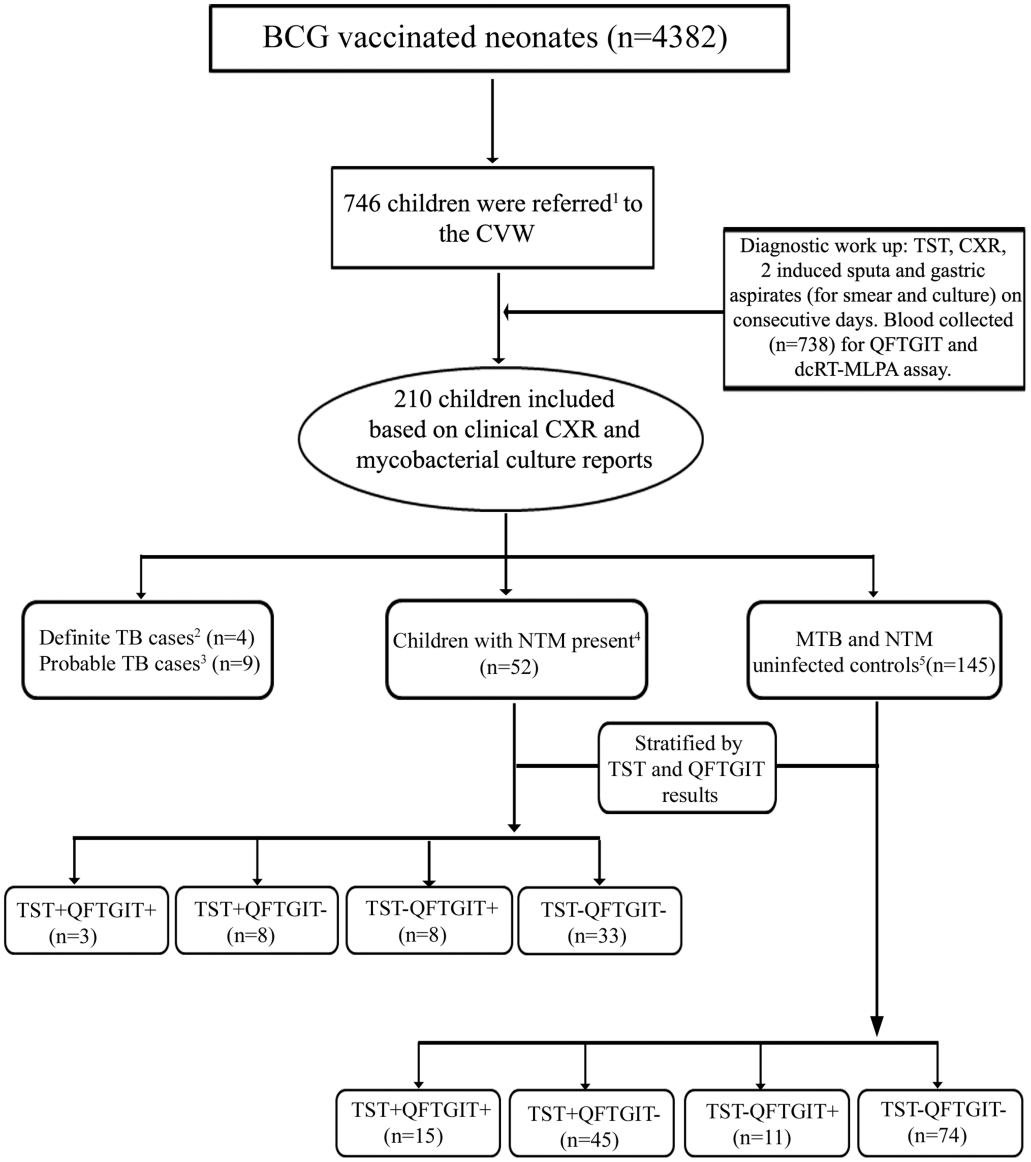
Flow chart of patients recruited to the study. ^1^Referral based on cough >2weeks, FTT, history of contact with a TB case and a TST ≥10 mm at study closure ^2^Abnormal CXR; MTB positive by the Hain MTB test or Roche test. ^3^Abnormal CXR; culture negative for MTB. ^4^Normal CXR; culture positive for NTM and ^5^Normal CXR; culture negative for MTB and NTM. Abbreviations: FTT – failure to thrive; CVW- case verification ward; CXR – chest X-ray; TST - tuberculin skin test; QFTGIT – QuantiFERON Gold In-tube test; dcRT-MLPA – dual colour reverse transcriptase – multiplex ligation dependent probe amplification; MTB – *Mycobacterium tuberculosis*; NTM – non-tuberculous mycobacteria.

For the purpose of this study, the 210 children were re-classified according to whether they had TB disease (n = 13) as previously defined, or no TB judged by culture negativity for MTB and a normal CXR, the latter group (no TB) were further subdivided as either NTM present (defined by ≥1 specimen culture positive for NTM; n = 52) or culture negative for MTB and NTM, referred to as controls (n = 145) (Fig. 1). Notably, none of the children with NTM present fulfilled the criteria for NTM disease suggested by the American Thoracic Society, NTM disease should be considered if there is (i) a compatible clinical presentation, (ii) a radiographic picture consistent with the diagnosis of NTM, (iii) exclusion of other diagnoses, and (iv) the recovered NTM species is present in sufficient quantities from consecutive specimens [5].

### Confirmation of NTM

Acid fast bacteria (AFB) culture positive samples were speciated by the HAIN kit (GenoType MTBC and CM), Hain Life Sciences, Germany). The HAIN CM kit identifies only 15 commonly isolated NTMs [17]. AFB culture positive samples that were identified as non-MTB complex mycobacteria, but which could not be further speciated by the HAIN CM kit are designated as NTM species in this study.

### Sample processing for immune biomarker analysis

For identifying biomarkers at the transcriptional level, a method which uses a pre-selected panel of genes, dual-colour reverse-transcriptase – multiplex-ligation-dependent-probe-amplification (dcRT-MLPA) was applied [18]. The genes in the panel consisted of 4 housekeeping genes, used as internal controls, and 45 genes identified as differentially expressed during MTB infection and/or disease in adults, by screening of different populations by qPCR and microarray [18]. Total RNA was extracted from PAXgene blood collection tubes (n = 210) using the ‘PAXgene Blood RNA kit’ (PreAnalytiX, Hilden, Germany) according to the manufacturer's instructions. RNA concentration and purity (A_260/280_ nm ratio) was measured using a spectrophotometer (Thermoscientific, Delaware, USA).

For the dcRT-MLPA experiment, 130–150 ng of total RNA was used. The dcRT-MLPA experimental protocol has been described in detail previously [15], [18]. The amplified PCR products were diluted 1∶10 with nuclease free H_2_O and added to a mixture of Hi-Di-Formamide with 400HD ROX size standard. The denatured (at 95°C for 5 min) products, were immediately cooled on ice. Fragment analysis was performed on a 3730 capillary sequencer (Life Technologies, California, USA), and the data imported into the Gene mapper software (Life Technologies, California, USA). The peak area data (arbitrary units) of replicates was averaged, normalized against *GAPDH*, and log2 transformed as described [18]. Of the 45 genes analyzed, 7 genes had expression levels below the cut off value of 7.64 (corresponding to a peak area <200 arbitrary units) and one gene CD14, co-localized with a primer-dimer peak and was therefore omitted from analysis.

For the identification of biomarkers at the translational level, supernatants from the QFTGIT assay (Nil and TB-ag tubes) (n = 210) were analyzed by a customized 10-plex cytokine/chemokine kit (Bio-Rad Laboratories Inc., California, USA). For data analysis, the cytokine/chemokine concentrations (pg/mL) in the Nil and TB-ag tubes were used and analyzed individually.

### Statistical analysis

Differences in biomarkers (as measured by dcRT-MLPA and the Bio-Plex assay) between groups were evaluated by non-parametric analysis (Mann-Whitney U test and Kruskal-Wallis test with Dunn's post-hoc test for multiple comparison) using IBM SPSS software version 21. A double sided p-value<0.05 was considered significant. GraphPad Prism 5 software was used for graphing the dot plots.

### Ethics approval

The study was conducted according to the Helsinki (4^th^ revision) declaration and approved by the institutional ethical review board of the St. John's Medical College and an independent ethics committee contracted by the Aeras Global TB Vaccine Foundation. At the time of participant enrollment a written informed consent was obtained from parents/guardians. This study was also approved by the Ministry of Health Screening Committee of the Government of India (No. 5/8/9/60/20006-ECD-I).

## Results

### Characteristics of study groups

The participants selected for this study were a subset of 210 children selected from a larger (n = 4382) longitudinal cohort study based on the availability of a full clinical workup and a full array of blood samples (Fig. 1). Baseline characteristics of the 210 children categorized by study groups are presented in Table 1. The gender distribution was similar between the groups. Children with NTM present had the same frequency of respiratory symptoms as controls, whereas as expected, children with TB disease had more respiratory symptoms than the other two groups (for both groups p = 0.03). Children with NTM present had less known exposure to TB (∼2%) than the other two groups, but more frequently had FTT (85%; p = 0.07). NTM were isolated from IS and GA samples with the same frequency, whereas MTB was only isolated from GA. NTM isolates (42.3%) that could not be identified at the species level by the HAIN CM kit were designated as *Mycobacterium species (M. spp.)*. The majority of NTMs that could be speciated by the HAIN test were: *Mycobacterium fortuitum* (40.4%) and *Mycobacterium intracellulare* (15.4%). About 3.0% of children were culture positive for NTMs on two consecutive days and samples that had the same NTM species cultured on both days were low (<1%) (Table 2).

**Table 1:** Baseline characteristics of 210 children.

Baseline characteristics	TB disease n = 13 (%)	Children with NTM present n = 52 (%)	Controls n = 145 (%)
**Age (months)**			
0–12 (n = 43)	4 (30.8)	5 (9.6)	34 (23.4)
13–24 (n = 135)	7 (53.8)	40 (76.9)	88 (60.7)
25–35 (n = 32)	2 (15.4)	7 (13.5)	23 (14.5)
**Gender**			
Male (n = 125)	7 (53.8)	28 (53.8)	90 (62.1)
Female (n = 85)	6 (46.2)	24 (46.2)	55 (37.9)
**CXR**			
Abnormal-TB (n = 11)	11 (84.6)	0 (0.0)	0 (0.0)
**Cough more than 2 weeks**			
Yes (n = 18)	4 (30.8)	3 (5.8)	11 (7.6)
**Failure to thrive**			
Yes (n = 154)	8 (61.5)	44 (84.6)	102 (70.3)
**History of contact with a TB case**			
Yes (n = 11)	1 (7.7)	1 (1.9)	9 (6.2)
**TST**			
Positive (n = 75)	4 (30.8)	11 (21.2)	60 (41.4)
**QFTGIT**			
Positive (n = 40)	3 (23.1)	11 (21.2)	26 (17.9)
**Smear/culture positivity for MTB and NTM**			
Only IS positive (n = 22)	0 (0.0)	22 (42.3)	0 (0.0)
Only GA positive (n = 24)	2 (15.4)	22 (42.3)	0 (0.0)
Both IS and GA positive (n = 10)	2 (15.4)	8 (15.4)	0 (0.0)

Abbreviations: CXR-Chest X-ray; TST- tuberculin skin test; QFTGIT- QuantiFERON In-tube TB Gold test; IS-Induced sputum; GA-gastric aspirates; MTB- *Mycobacterium tuberculosis*; NTM-non-tuberculous mycobacteria.

**Table 2:** Day 1 and Day 2 induced sputum (IS) and gastric aspirate (GA) culture positive results among 210 study participants.

NTM species	NTM isolation rates per study participant (%)
*M. spp.*	19 (9.0%)
*M. fortuitum*	16 (7.6%)
*M. intracellulare*	6 (2.9%)
*M. scrofulaceum*	2 (1.0%)
*M. kansasi*	1 (0.5%)
*M. avium*	1 (0.5%)
*M. abscessus*	1 (0.5%)
* *M. fortuitum, M. intracellulare*	1 (0.5%)
* *M. fortuitum, M. kansasi*	1 (0.5%)
* *M. fortuitum, M. scrofulaceum*	1 (0.5%)
* *M. fortuitum, M. spp.*	1 (0.5%)
* *M. intracellulare, M. spp.*	1 (0.5%)
* *M. fortuitum, M. intracellulare, M. spp.*	1 (0.5%)
Total number of children culture positive for NTM	**52 (24.8%)**

*Mixed NTM species were isolated from day 1 and day 2 IS and GA samples.

### Biomarker profiles in children with NTM present compared to controls

**Figure 2:**
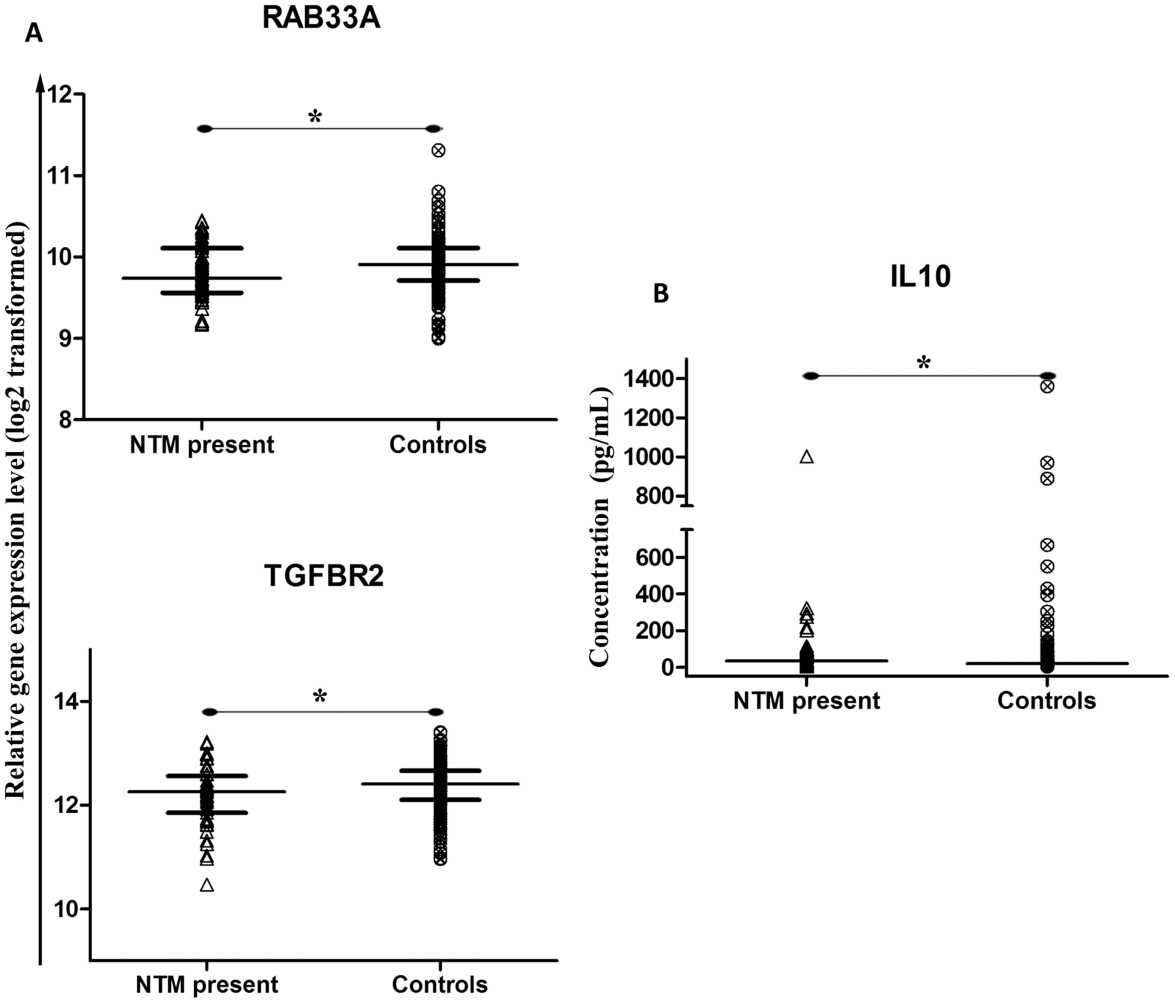
Dot-plot graph depicting genes and proteins that are differentially expressed between children with NTM present and children that were culture negative for MTB and NTM. (A) The median with inter quartile range relative gene expression (log 2 transformed) of genes from peripheral blood. (B) The median concentration (pg/mL) of cytokines in the QFTGIT supernatants of whole blood without stimulation. p-value<0.05 (*) was considered to be significant.

We first assessed the effect of the presence of NTM on immune biomarkers in the presumed target population for TB booster vaccines: BCG-vaccinated children without TB disease. Of 45 biomarkers tested (Table S1), there was no appreciable change for most, but transcription of mRNA for *RAB33A* and *TGFBR2* was down-regulated (p<0.05) in children with NTM present (n = 52) compared to controls (Fig. 2a). Bio-plex analysis on unstimulated QFTGIT supernatants (Nil tube) showed that compared to controls, the expression of cytokine IL-10 (p<0.05) was up-regulated in children with NTM present (Fig. 2 b).

### Comparison of biomarker profiles between children with TB disease, NTM present and controls

**Figure 3:**
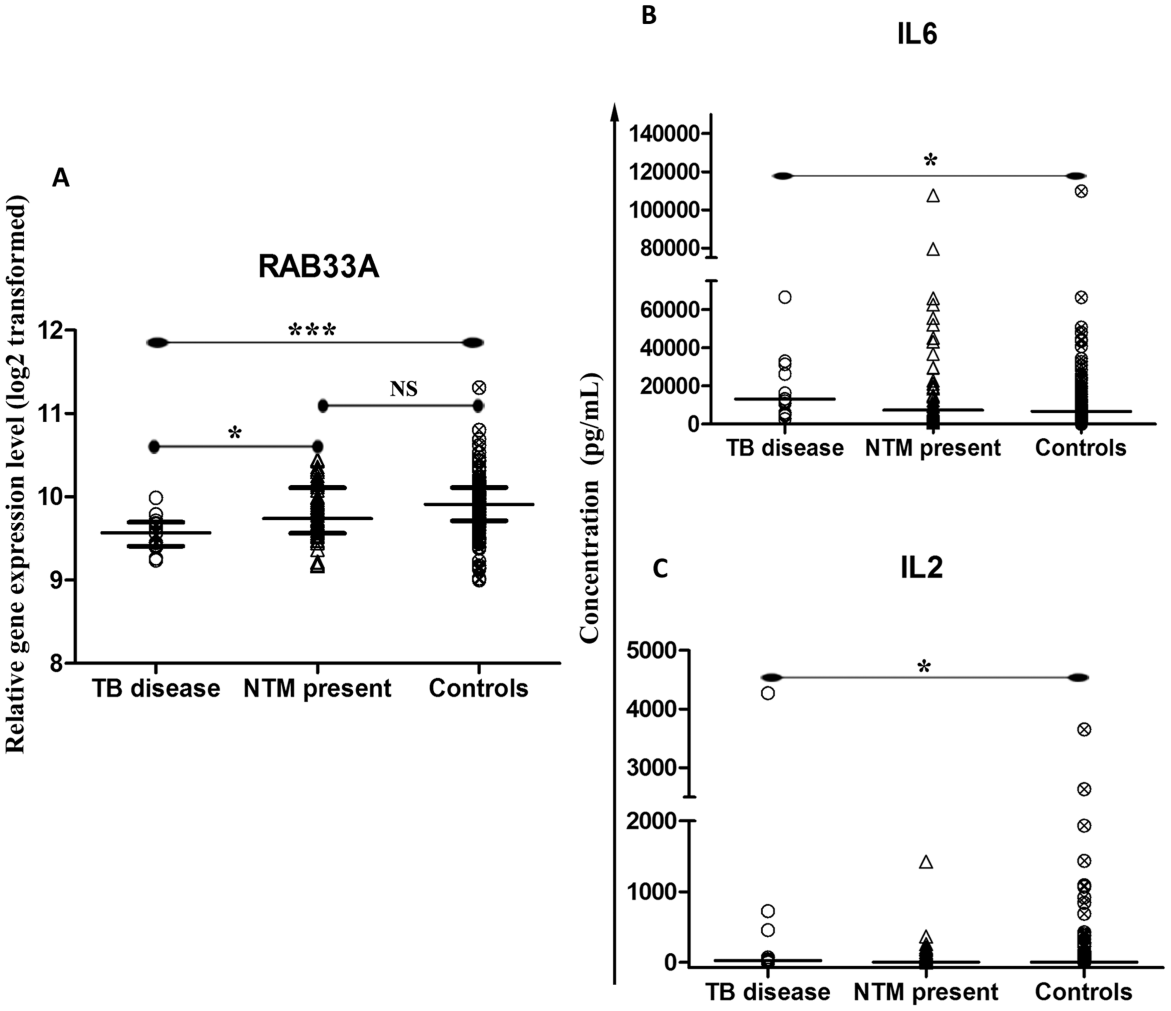
Dot-plot graph depicting genes and proteins that are differentially expressed between the three clinical groups: TB disease, NTM present and controls. (A) The median with inter quartile range relative gene expression (log 2 transformed) of genes from peripheral blood. (B) The median concentration (pg/mL) of cytokines in the QFTGIT supernatants of whole blood without stimulation. (C) The median concentration (pg/mL) of cytokines in the QFTGIT supernatants after stimulation of whole blood with *M. tuberculosis* antigens. p-value<0.05 (*), <0.01 (**), <0.001 (***) were considered to be significant; NS - not significant.

We next assessed the potential effect of the presence of NTM on biomarkers in a TB diagnostic setting. Compared to controls (n = 145) and children with NTM present (n = 52), the direct *ex vivo* transcription of *RAB33A* was down-regulated (p<0.001; p<0.05, respectively) in children with TB disease (n = 13; Fig. 3a). Furthermore, Bio-plex analysis on unstimulated whole blood QFTGIT supernatants (Nil tube) showed that the expression of cytokine IL-6 was up-regulated in TB disease (p<0.05) compared to controls (Fig. 3b). Similarly, the analysis from stimulated whole blood QFTGIT supernatants (TB-ag tube) showed that the expression of cytokine IL-2 was up-regulated (p<0.05) in children with TB disease compared to controls (Fig. 3c). Interestingly, these differences between children with TB disease and controls were not evident in our earlier study [15], when children with TB disease were compared to controls (TST and QFTGIT negative children), presumably because 33 of 107 of these controls had NTM present.

**Figure 4:**
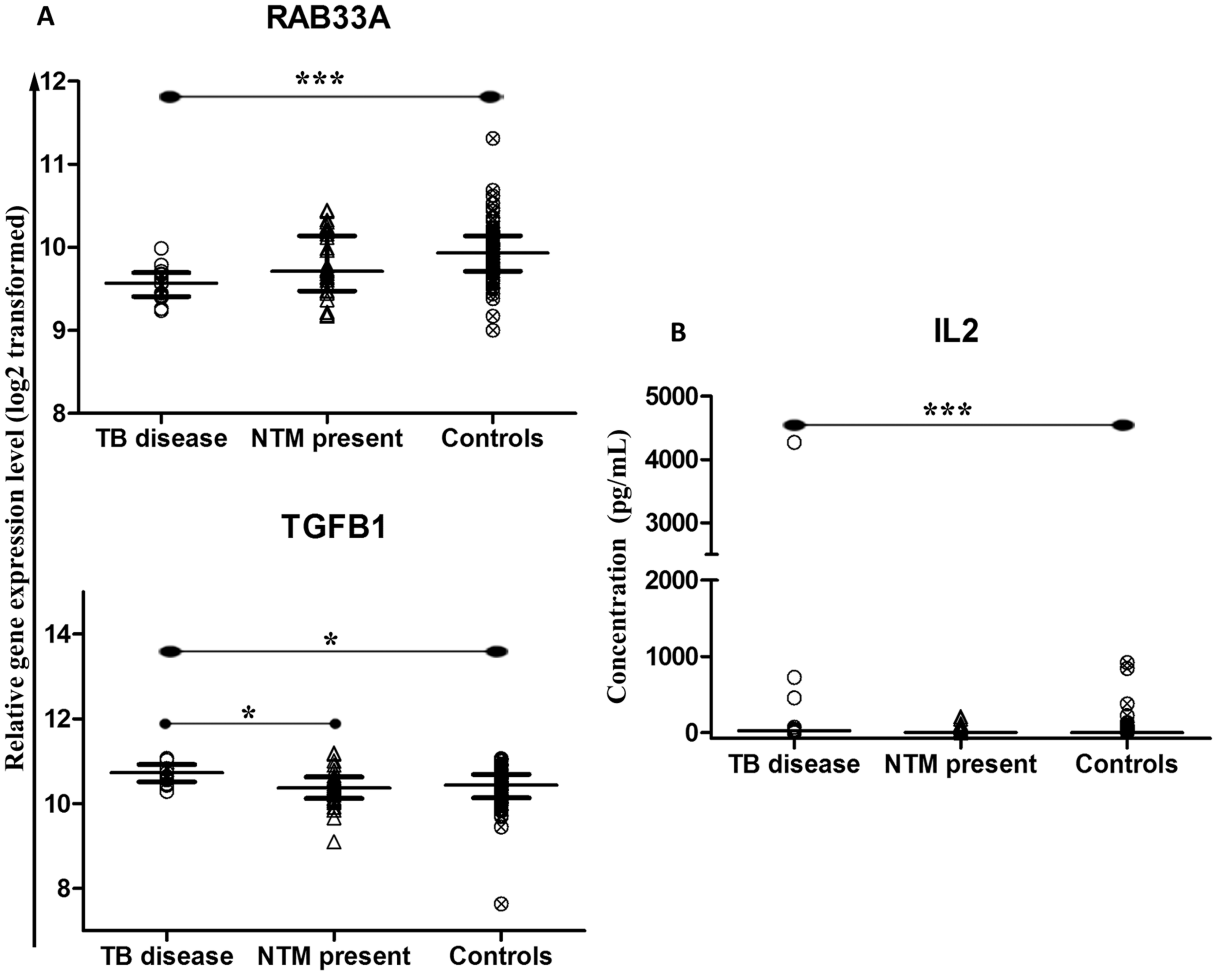
Dot-plot graph depicting genes and proteins that are differentially expressed between the three clinical groups: TB disease, NTM present and controls (defined as MTB, NTM uninfected children and negative for TST and QFTGIT). (A) The median with inter quartile range relative gene expression (log 2 transformed) of genes from peripheral blood. (B) The median concentration (pg/mL) of cytokines in the QFTGIT supernatants after stimulation of whole blood with *M. tuberculosis* antigens. p-value<0.05 (*), <0.01 (**), <0.001 (***) were considered to be significant.

In the analyses above, the groups of children with NTM present and controls contained children with divergent results for TST and QFTGIT. Children with positive TST and/or QFTGIT tests may have latent TB infection. This is likely to have increased the immunological heterogeneity within these groups. We therefore, repeated the analyses above with “cleaner” groups consisting of TST and QFT negative children only: children with NTM present (n = 33); and controls (n = 74). This sub-analysis identified the same differences as above with regard to a down-regulation of *RAB33A* (p<0.001) and an up-regulation of IL-2 (p<0.001) in children with TB disease (Fig. 4a and 4b). In addition, this analysis also revealed an up-regulated transcription of *TGFβ1* (p<0.05) in children with TB disease compared to the other two groups (for both p<0.05) (Fig. 4a).

## Discussion

In the context of TB disease management, a likely impact of NTM on the TB protection induced by the BCG vaccine is well recognized although the mechanisms are unclear. BCG is used as the “gold standard” for induction of protective immune responses against TB in humans, however, there is consensus that it does not induce complete protection against TB in any animal species [19]. Also, clinical trials have shown varying efficacy of the BCG vaccine, and multiple reasons have been suggested, including a potential role for NTM exposure [20]. The immuno-modulating properties of NTM are also likely to affect studies of TB-diagnostic biomarkers as well as immuno-correlates of TB protection by which it is hoped the efficacy of new TB vaccines can be evaluated [21].

In the present study of children, all BCG-vaccinated at birth and aged <3 years, we show that the genes *TGFBR2, RAB33A* and the cytokine IL-10 were differentially expressed in children with NTM-positive cultures compared to controls. Background exposure of NTM in the setting of a vaccine trial might therefore interfere with these markers if used as correlates of protection. *RAB33A* is a member of small guanosine triphosphatase (GTPase) family and is involved in vesicle transport and fusion [22]. Dysregulation of GTPases has shown to play a role in blocking the phagosome maturation [23] which is a major survival strategy for MTB [24]. *TGFBR2* is involved in signal transduction and mediating inhibition of cell growth and induction of cell death [25], [26]. IL-10 is an anti-inflammatory cytokine which in the setting of MTB infection inhibits CD4 T-cell responses and dendritic cell functions [27].

We and others have shown that, *RAB33A* seems to have a potential as a diagnostic marker of TB disease [15], [28], [29]. With this study we add that the expression of *RAB33A* is reduced in children with TB disease compared to children without TB regardless of TST/QFTGIT results or NTM presence. When restricting the comparison of children with NTM present to those with a negative TST and QFTGIT result (to control for potential effects of MTB infection), we found no significant difference in the transcription of *RAB33A* between children with TB disease and those with NTM present. However, the median value for NTM-positive children consistently lay between that of the TB cases and the mycobacteria-negative children so this result may reflect the smaller sample size of this group or that down-regulation of *RAB33A* is more strongly impacted by disease, rather than carriage/infection with mycobacteria. Nevertheless, the reduced transcription of *RAB33A* in children with NTM present compared to controls raises the possibility of an impact of NTM presence on the specificity if *RAB33A* were to be used in a diagnostic setting. Furthermore, as we have published earlier *TGFβ1* appears to be up-regulated in children with TB disease compared to MTB uninfected children [15]. This study provides evidence that *TGFβ1* is up-regulated in children with TB disease regardless of NTM presence, but only in TST and QFTGIT negative children, suggesting that MTB infection may also be modulating expression of this gene, but that NTM exposure does not. In contrast, increased levels of IL-2 and IL-6 in children with TB disease was only seen compared to MTB negative controls and not compared to children with NTM present, suggesting a potential interference of NTM on these read-outs in a diagnostic setting. TGF-β1 performs many cellular functions and is involved in wound healing of granulomatous lesions in TB [30]. IL-2 promotes T cell replication and is essential for maintaining adaptive cellular immunity and granuloma formation [31]. The cytokine IL-6 is produced by the innate immune cells early following **a** pathogen encounter and is implicated in the host inflammatory response to MTB [27].

In a study from South Africa, NTM were isolated in 6% of all children investigated for pulmonary TB and association of NTM isolation with constitutional symptoms was suggestive of host recognition [14]. In the present study, NTM were isolated in about a quarter of the infants in this study. This is a relatively high proportion, but the lack of pathology seen in CXR in children with NTM present and the lack of associated symptoms suggest no association with disease. The possibility of laboratory contamination was considered minimal, due to strict adherence to sampling and laboratory procedures including internal and external quality control. Moreover, the present study shows that NTM were less likely to be isolated from clinical samples at younger ages 0–12 months (adjusted for gender and symptoms; OR 0.18, CI 0.04–0.79) suggesting a reduced interaction with the environment in younger children, an unlikely finding if NTM presence was caused by contamination, since NTM are ubiquitously found in soil and water. A possible limitation of this study is that we were not able to determine the background NTM rate in a control group of children. Children were referred for investigation if they were considered to be at risk of TB, due to suspected illness or history of TB contact, thereby introducing an ascertainment bias. This factor was partly overcome by comparing children with culture-confirmed NTM or MTB only. Exposure to NTM through the oral or respiratory route is usually asymptomatic. However, our study shows that NTM carriage or transient and likely repeated exposure elicits responses which resemble the response seen in MTB infection [15], [32]. This highlights the importance of evaluation of TB biomarkers in the context of exposure to NTM.

In conclusion, it is clear that NTM presence modulates host immunity. Even though NTM exposure rarely causes a symptomatic infection in healthy individuals, this study shows that NTM carriage or transient and likely repeated exposure does elicit some of the same immune responses as MTB infection, namely down-regulation of and up-regulation of *TGFβ1*. In different settings and populations, these immune biomarkers have shown a potential as discriminatory diagnostic biomarkers in MTB infection and disease. Whether these markers hold a potential as correlates of TB protection remains to be elucidated. Nevertheless, the results from the present study suggest that NTM presence should be considered when evaluating future biomarkers for this purpose, as the presence of NTM may impact the specificity of immune biomarkers for TB outcomes.

## Supporting Information

Checklist S1 STROBE Checklist.
(DOC)

Supporting Information S1 Preparing for TB vaccine efficacy trials, Palamaner field site, Chittoor district, Southern India. Studies on baseline epidemiology, mycobacterial diversity, improved diagnosis, biomarkers of protection and phase I trials, conducted by the TB Trials Study Group. Picture courtesy TB Trials Study Group.
(PPTX)

Table S1 Genes investigated in the dcRT-MLPA and their functions.
(DOCX)
