# Supplementary figures and images for: Effect of Non-tuberculous Mycobacteria on Host Biomarkers Potentially Relevant for Tuberculosis Management
Source: PLoS Negl Trop Dis. 2014 Oct 16;8(10):e3243. doi: 10.1371/journal.pntd.0003243 (PMC4199571; doi:10.1371/journal.pntd.0003243)

## Slide 1
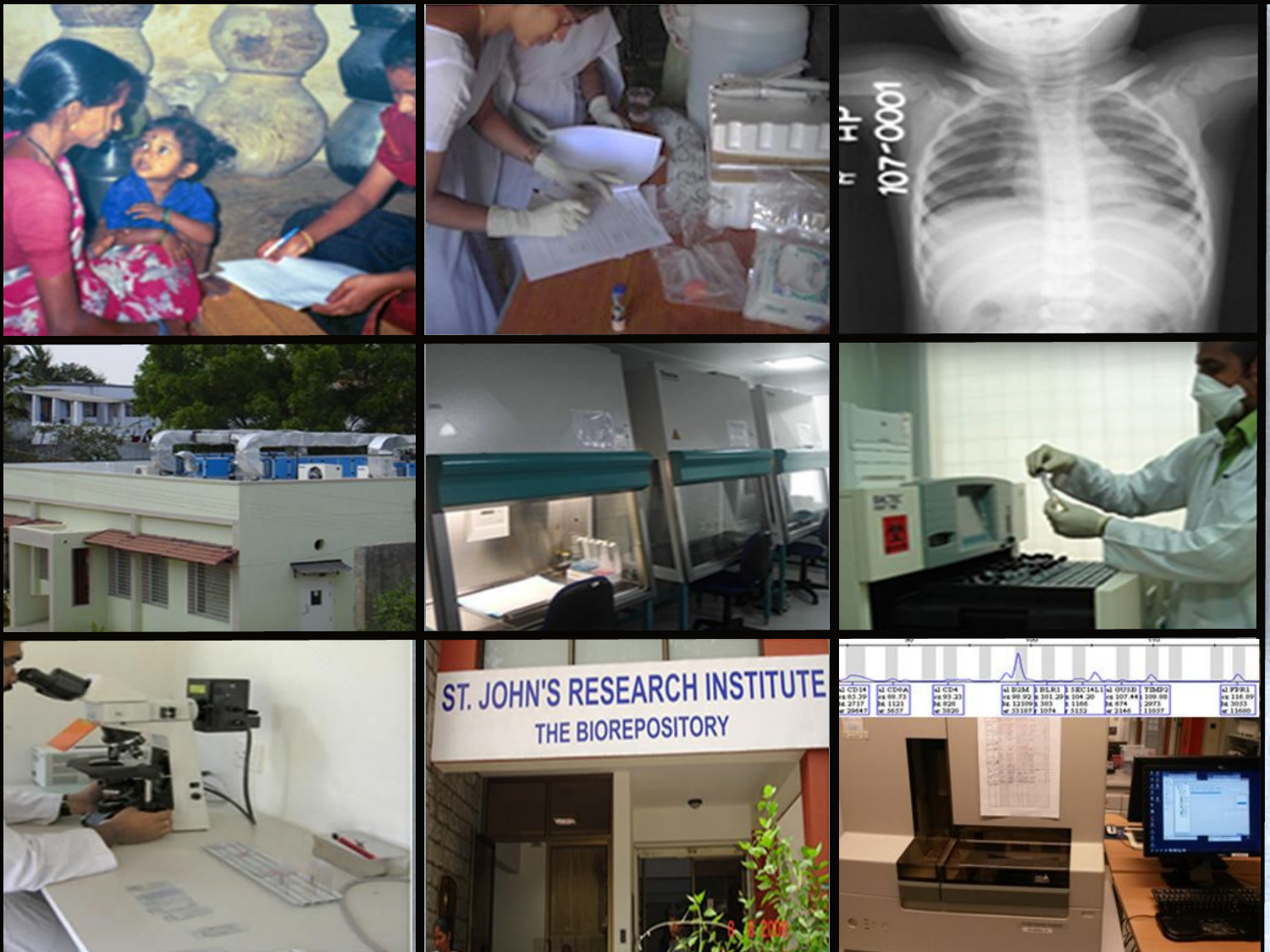

Supplement: Supporting Information S1 — Preparing for TB vaccine efficacy trials, Palamaner field site, Chittoor district, Southern India. Studies on baseline epidemiology, mycobacterial diversity, improved diagnosis, biomarkers of protection and phase I trials, conducted by the TB Trials Study Group. Picture courtesy TB Trials Study Group. (PPTX) [file pntd.0003243.s002.pptx]
