# Supplementary material for: Effect of Non-tuberculous Mycobacteria on Host Biomarkers Potentially Relevant for Tuberculosis Management
Source: PLoS Negl Trop Dis. 2014 Oct 16;8(10):e3243. doi: 10.1371/journal.pntd.0003243 (PMC4199571; doi:10.1371/journal.pntd.0003243)
Supplement: Table S1 — Genes investigated in the dcRT-MLPA and their functions. (DOCX) [file pntd.0003243.s003.docx]

Table S1: Genes investigated in the dcRT-MLPA and their functions

| Genes | Putative role | Reference |
| --- | --- | --- |
| BCL2 | B-cell lymphoma 2, regulates cell death (apoptosis). | [1] |
| BLR1 | Belongs to CXC chemokine receptor family (CXCR5), plays a vital role in T cell localization within TB granulomas  and defence against MTB infection. | [2] |
| BPI | Bactericidal permeability-increasing protein, found in neutrophil granules of and associated with host defence. | [3] |
| CASP8 | Cysteine-aspartic acid protease family, plays a central role in the execution-phase of cell apoptosis. | [1] |
| CCL4 | Chemokine (c-c motif) ligand 4 or Macrophage inflammatory protein 1β (MIP-1β), acts as chemo-attractant for a variety of immune cells. | [4] |
| CCL13 | Chemokine (c-c motif) ligand 13, plays a dominant role in homing of lymphocytes and dendritic cells,  contributes to control of MTB infection. | [5] |
| CCL19 | Chemokine (c-c motif) ligand 19, as similar function to CCL13. | [5] |
| CCL22 | Belongs to CC chemokine family, plays a role in the trafficking of activated T lymphocytes to inflammatory sites. | [4] |
| CCR7 | CC chemokine receptor 7, mediates trafficking of dendritic cells from the lungs to the mediastinal lymph node during TB disease. | [6] |
| CD3E | T-cell glycoprotein CD3 epsilon, plays a role in intracellular signal-transduction pathways. | [7] |
| CD4 | Cluster of differentiation 4, plays a major role in adaptive immunity to TB. | [8] |
| CD8A | Cluster of differentiation 8A, identifies cytotoxic T- cells that interact with MHC class I targets and  contributes to control of MTB infection. | [9] |
| CD14 | Cluster of differentiation 14, a component of the innate immune system and acts a co-receptor for recognizing MTB. | [10] |
| CD19 | Cluster of differentiation 19, a B cell marker and essential for B cell activation. | [11] |
| CD163 | Cluster of differentiation 163, a hemoglobin scavenger receptor and is exclusively expressed in macrophages on inflammation. | [12] |
| CTLA4 | Cytotoxic T-lymphocyte antigen 4, plays a role in the regulation of T cell immune responses and  involved in the maintenance of T cell homeostasis. | [13] |
| CXCL10 | CXC chemokine 10 or interferon gamma-induced protein 10 (IP-10), secreted by number of types in response to IFN-γ  and involved in stimulation of natural killer cells and T-cell migration in MTB infection. | [14] |
| FASLG | Fas ligand, a type II transmembrane protein, belonging to TNF family and induces apoptosis. | [1] |
| FCGR1A | Fc region of immunoglobulin gamma functions in both innate and adaptive immune responses. | [15] |
| FOXP3 | Forkhead box P3, belongs to the transcription factor family and is involved in various cellular processes,  acts as an important regulator for T-cell development. | [16] |
| FPR1 | Formyl peptide receptor 1, a member of G-protein-coupled receptor family and plays a vital role in the innate immune system. | [17] |
| IFNγ | Interferon gamma, secreted by several cell types such as NK cells, CD4^+^ and CD8^+^ T-cells, contributes to control of MTB infection. | [18] |
| IL2RA | Interleukin 2 receptor gene, plays an important role in the control of immune system response. | [19] |
| IL4 | Interleukin 4, induces Th2 responses and plays some role in the defense against MTB infection. | [18] |
| IL4d2 | IL-4 antagonist and splice variant of interleukin-4. | [20] |
| IL7R | Interleukin-7 receptor protein, plays a role in the development of immune cells. | [21] |
| IL10 | Interleukin-10, an anti-inflammatory cytokine and may contribute to TB pathogenesis. | [18] |
| IL22RA1 | Interleukin 22 receptor alpha 1belongs to class II cytokine receptor family and activates various signaling pathways. | [22] |
| LAG3 | Lymphocyte-activation gene 3, an important regulatory molecule involved in expansion of activated T-cells. | [23] |
| LTF | Lacto-transferrin also called as Lactoferrin (LF), a secreted mediator that connects innate and adaptive immune response. | [24] |
| MARCO | Macrophage receptor, essential for immune response to bacterial infections and mediates phagocytosis of pathogens. | [25] |
| MMP9 | Matrix metallo-peptidase 9, induced by MTB infection and has a role with MCP-1in recruiting macrophages to the lungs during granuloma formation. | [26] |
| NCAM1 | Neural cell adhesion molecule 1, mediates several intracellular signaling pathways. | [27] |
| RAB13 | Ras related protein-13, a small GTPase family member, regulates assembly of functional tight junctions in epithelial cells. | [28] |
| RAB24 | Ras related protein-24, a small GTPase family member, regulates intracellular protein trafficking between  endoplasmic reticulum and cis-Golgi compartment. | [28] |
| RAB33A | Ras related protein-33A, a small GTPase family member, dysregulation of GTPase plays a role in blocking of phagosome maturation,  which is a major survival strategy for MTB. | [29] |
| SEC14L1 | SEC14 cytosolic factor family plays a role in the intracellular transport system. | [30] |
| SPP1 | Secreted phosphoprotein 1, acts as a cytokine that up-regulates the expression of interferon-gamma and interleukin-12. | [31] |
| TGFB1 | Transforming growth factor β1, an anti-inflammatory cytokine, performs many cellular functions and involved in  wound healing of granulomatous lesions in TB. | [32] |
| TGFBR2 | Transforming growth factor β receptor 2, involved in signal transduction and response to inhibit cell growth and division. | [33] |
| TIMP2 | Tissue inhibitor of metallo-proteinases, involved in pathological changes and pathogenesis of pulmonary TB. | [10] |
| TNF | Tumor necrosis factor, a cytokine that plays multiple roles in the immunopathology of TB and is essential for controlling MTB infection. | [18] |
| TNFRSF1A | TNF receptor superfamily member1A, mediates apoptosis and functions as a regulator of inflammation. | [34] |
| TNFRSF1B | TNF receptor superfamily member 1B, mediates anti-apoptotic signals. | [34] |
| TNFRSF18 | TNF receptor superfamily member 18, involved in T-cell activation and programmed cell death. | [16] |
| ABR | Active BCR-Related gene contains a GTPase-activating protein domain (used as endogenous control). |  |
| B2M | β2 microglobulin, a component of MHC class I molecules (used as endogenous control). |  |
| GAPDH | Glyceraldehyde 3-phosphate dehydrogenase, involved in quite a few non-metabolic processes (used as endogenous control). |  |
| GUSB | Glucuronidase β, regulates lysosomal storage function and co-regulated in response to stress (used as endogenous control). |  |

**References for Table S1**

1. Elmore S (2007) Apoptosis: a review of programmed cell death. Toxicol Pathol 35: 495-516.

2. Slight SR, Rangel-Moreno J, Gopal R, Lin Y, Fallert Junecko BA, et al. (2013) CXCR5(+) T helper cells mediate protective immunity against tuberculosis. J Clin Invest 123: 712-726.

3. Schultz H, Weiss JP (2007) The bactericidal/permeability-increasing protein (BPI) in infection and inflammatory disease. Clin Chim Acta 384: 12-23.

4. Le Y, Zhou Y, Iribarren P, Wang J (2004) Chemokines and chemokine receptors: their manifold roles in homeostasis and disease. Cell Mol Immunol 1: 95-104.

5. Khader SA, Rangel-Moreno J, Fountain JJ, Martino CA, Reiley WW, et al. (2009) In a murine tuberculosis model, the absence of homeostatic chemokines delays granuloma formation and protective immunity. J Immunol 183: 8004-8014.

6. Olmos S, Stukes S, Ernst JD (2010) Ectopic activation of Mycobacterium tuberculosis-specific CD4+ T cells in lungs of CCR7-/- mice. J Immunol 184: 895-901.

7. Banner B, Spicer Z, Alroy J (2003) Expression of CD3 epsilon subunit in gastric parietal cells: a possible role in signal transduction? Pathol Res Pract 199: 137-143.

8. Joosten SA, Goeman JJ, Sutherland JS, Opmeer L, de Boer KG, et al. (2012) Identification of biomarkers for tuberculosis disease using a novel dual-color RT-MLPA assay. Genes Immun 13: 71-82.

9. Axelsson-Robertson R, Weichold F, Sizemore D, Wulf M, Skeiky YA, et al. (2010) Extensive major histocompatibility complex class I binding promiscuity for Mycobacterium tuberculosis TB10.4 peptides and immune dominance of human leucocyte antigen (HLA)-B*0702 and HLA-B*0801 alleles in TB10.4 CD8 T-cell responses. Immunology 129: 496-505.

10. Wang C, Li YY, Li X, Wei LL, Yang XY, et al. (2014) Serum complement C4b, fibronectin, and prolidase are associated with the pathological changes of pulmonary tuberculosis. BMC Infect Dis 14: 52.

11. Depoil D, Fleire S, Treanor BL, Weber M, Harwood NE, et al. (2008) CD19 is essential for B cell activation by promoting B cell receptor-antigen microcluster formation in response to membrane-bound ligand. Nat Immunol 9: 63-72.

12. Moestrup SK, Moller HJ (2004) CD163: a regulated hemoglobin scavenger receptor with a role in the anti-inflammatory response. Ann Med 36: 347-354.

13. McCoy KD, Le Gros G (1999) The role of CTLA-4 in the regulation of T cell immune responses. Immunol Cell Biol 77: 1-10.

14. Lande R, Giacomini E, Grassi T, Remoli ME, Iona E, et al. (2003) IFN-alpha beta released by Mycobacterium tuberculosis-infected human dendritic cells induces the expression of CXCL10: selective recruitment of NK and activated T cells. J Immunol 170: 1174-1182.

15. Nigam A, Priya S, Bajpai P, Kumar S (2014) Cytogenomics of hexavalent chromium (Cr(6+)) exposed cells: A comprehensive review. Indian J Med Res 139: 349-370.

16. Koukouikila-Koussounda F, Ntoumi F, Ndounga M, Tong HV, Abena AA, et al. (2013) Genetic evidence of regulatory gene variants of the STAT6, IL10R and FOXP3 locus as a susceptibility factor in uncomplicated malaria and parasitaemia in Congolese children. Malar J 12: 9.

17. Zhou C, Zhou Y, Wang J, Feng Y, Wang H, et al. (2013) V101L of human formyl peptide receptor 1 (FPR1) increases receptor affinity and augments the antagonism mediated by cyclosporins. Biochem J 451: 245-255.

18. Raja A (2004) Immunology of tuberculosis. Indian J Med Res 120: 213-232.

19. Milani P, Marilley M, Sanchez-Sevilla A, Imbert J, Vaillant C, et al. (2011) Mechanics of the IL2RA gene activation revealed by modeling and atomic force microscopy. PLoS One 6: e18811.

20. Wassie L, Demissie A, Aseffa A, Abebe M, Yamuah L, et al. (2008) Ex vivo cytokine mRNA levels correlate with changing clinical status of ethiopian TB patients and their contacts over time. PLoS One 3: e1522.

21. Akashi K, Kondo M, Weissman IL (1998) Role of interleukin-7 in T-cell development from hematopoietic stem cells. Immunol Rev 165: 13-28.

22. Lim C, Savan R The Role of the IL-22/IL-22R1 Axis in Cancer. Cytokine & Growth Factor Reviews.

23. Sega EI, Leveson-Gower DB, Florek M, Schneidawind D, Luong RH, et al. (2014) Role of lymphocyte activation gene-3 (Lag-3) in conventional and regulatory T cell function in allogeneic transplantation. PLoS One 9: e86551.

24. Siqueiros-Cendon T, Arevalo-Gallegos S, Iglesias-Figueroa BF, Garcia-Montoya IA, Salazar-Martinez J, et al. (2014) Immunomodulatory effects of lactoferrin. Acta Pharmacol Sin 35: 557-566.

25. Komine H, Kuhn L, Matsushita N, Mule JJ, Pilon-Thomas S (2013) Examination of MARCO activity on dendritic cell phenotype and function using a gene knockout mouse. PLoS One 8: e67795.

26. Taylor JL, Hattle JM, Dreitz SA, Troudt JM, Izzo LS, et al. (2006) Role for matrix metalloproteinase 9 in granuloma formation during pulmonary Mycobacterium tuberculosis infection. Infect Immun 74: 6135-6144.

27. Hortsch M, Umemori H (2009) The sticky synapse: cell adhesion molecules and their role in synapse formation and maintenance. New York: Springer. xii, 453 p. p.

28. Jacobsen M, Repsilber D, Gutschmidt A, Neher A, Feldmann K, et al. (2005) Ras-associated small GTPase 33A, a novel T cell factor, is down-regulated in patients with tuberculosis. J Infect Dis 192: 1211-1218.

29. Doherty M, Wallis RS, Zumla A, group WH-TDRECjec (2009) Biomarkers for tuberculosis disease status and diagnosis. Curr Opin Pulm Med 15: 181-187.

30. Ribeiro FM, Ferreira LT, Marion S, Fontes S, Gomez M, et al. (2007) SEC14-like protein 1 interacts with cholinergic transporters. Neurochem Int 50: 356-364.

31. Renkl AC, Wussler J, Ahrens T, Thoma K, Kon S, et al. (2005) Osteopontin functionally activates dendritic cells and induces their differentiation toward a Th1-polarizing phenotype. Blood 106: 946-955.

32. Toossi Z, Gogate P, Shiratsuchi H, Young T, Ellner JJ (1995) Enhanced production of TGF-beta by blood monocytes from patients with active tuberculosis and presence of TGF-beta in tuberculous granulomatous lung lesions. J Immunol 154: 465-473.

33. Bellam N, Pasche B (2010) Tgf-beta signaling alterations and colon cancer. Cancer Treat Res 155: 85-103.

34. Croft M (2009) The role of TNF superfamily members in T-cell function and diseases. Nat Rev Immunol 9: 271-285.
